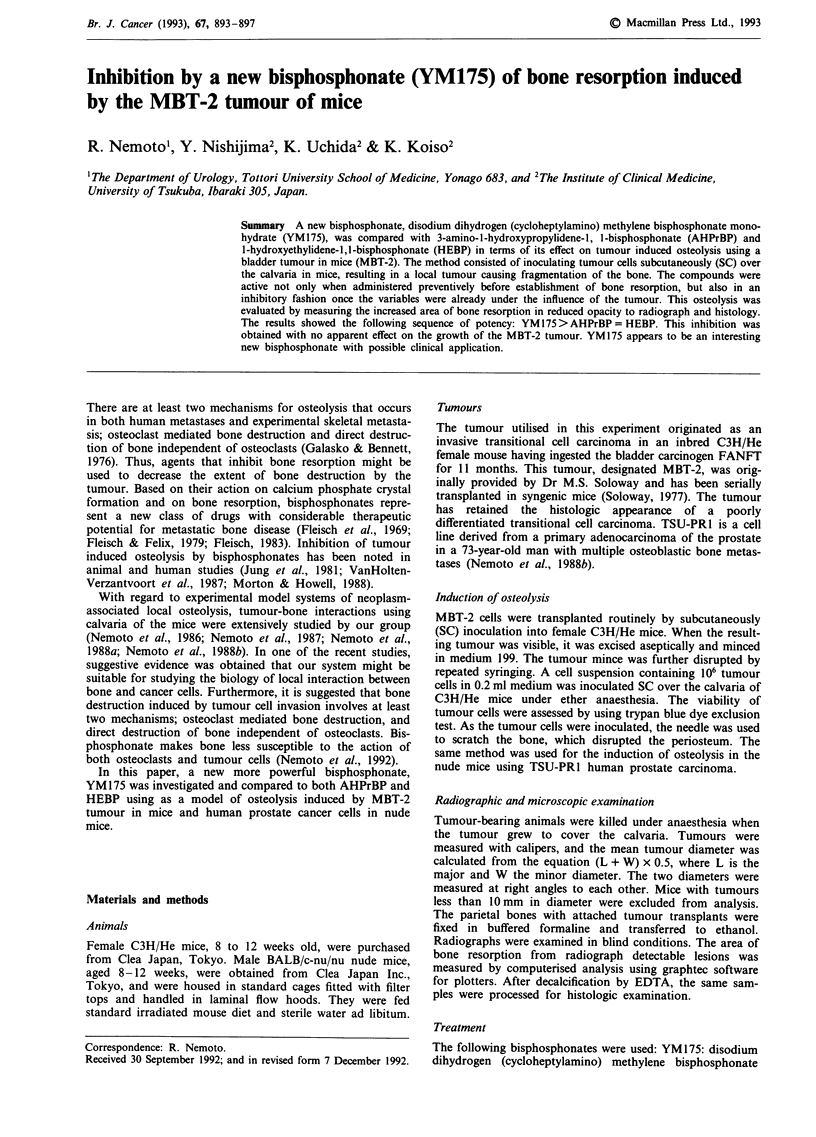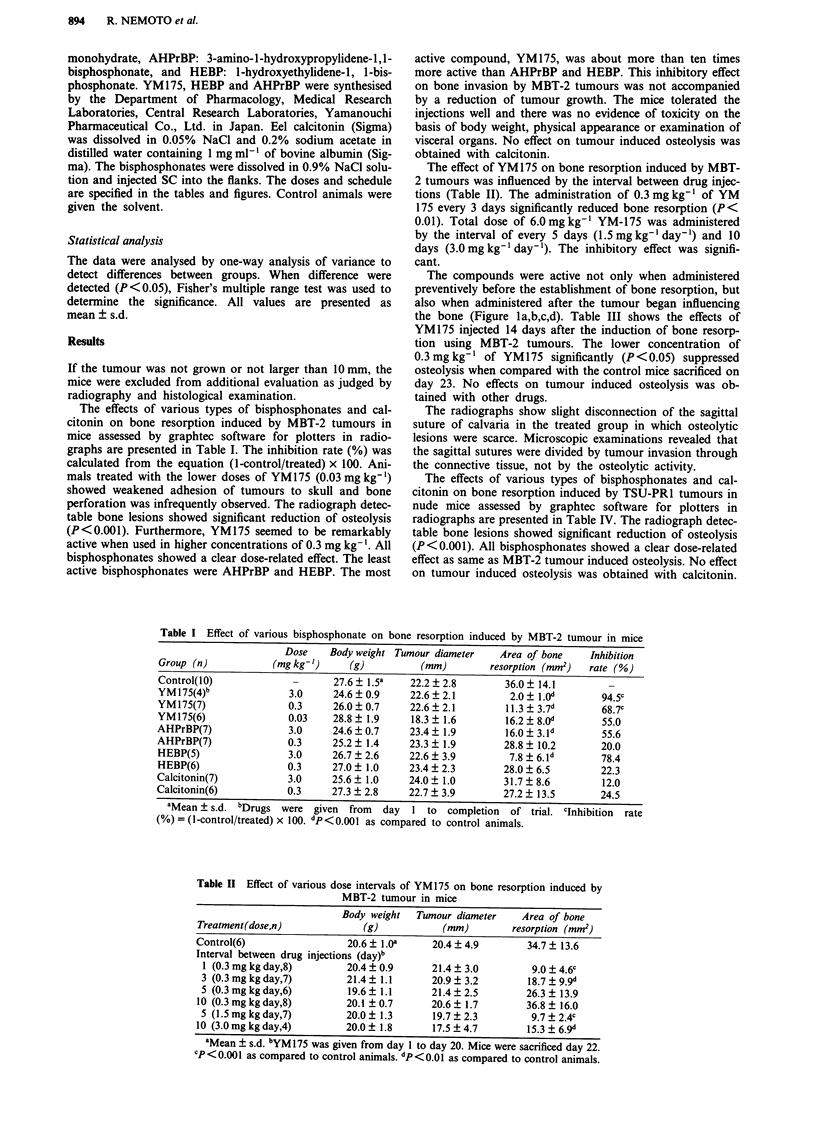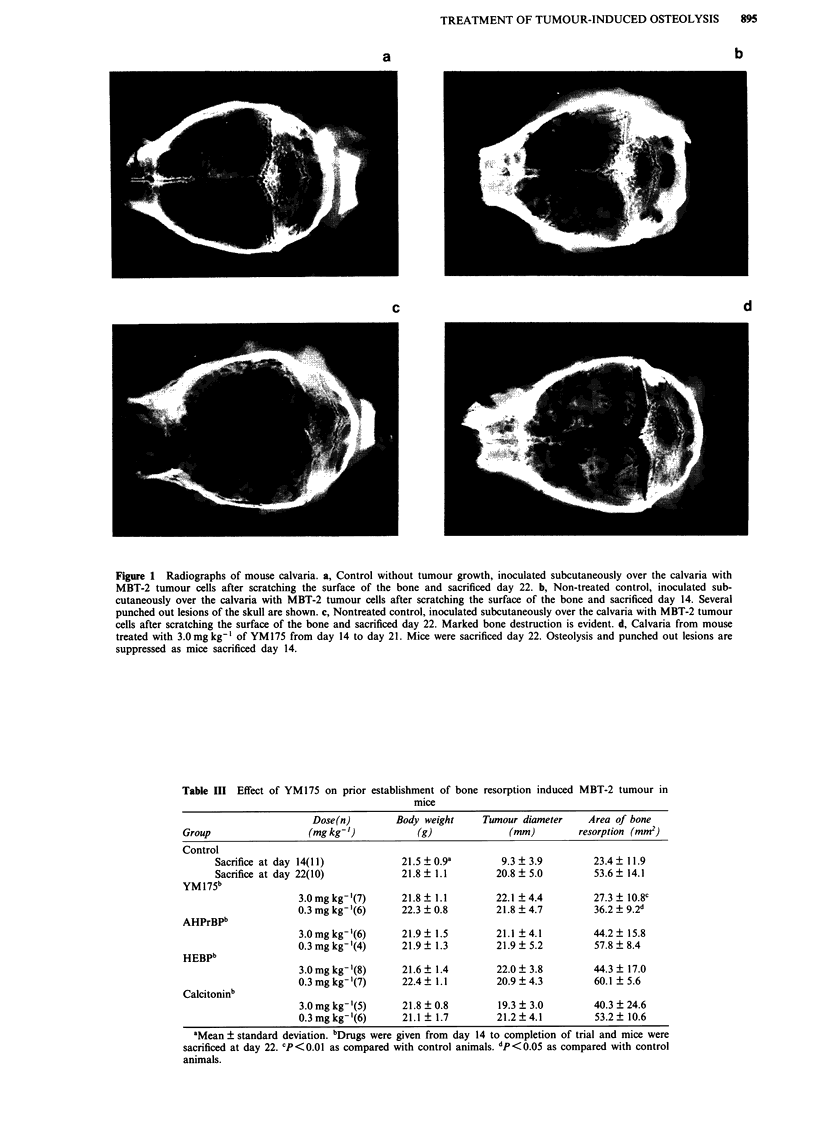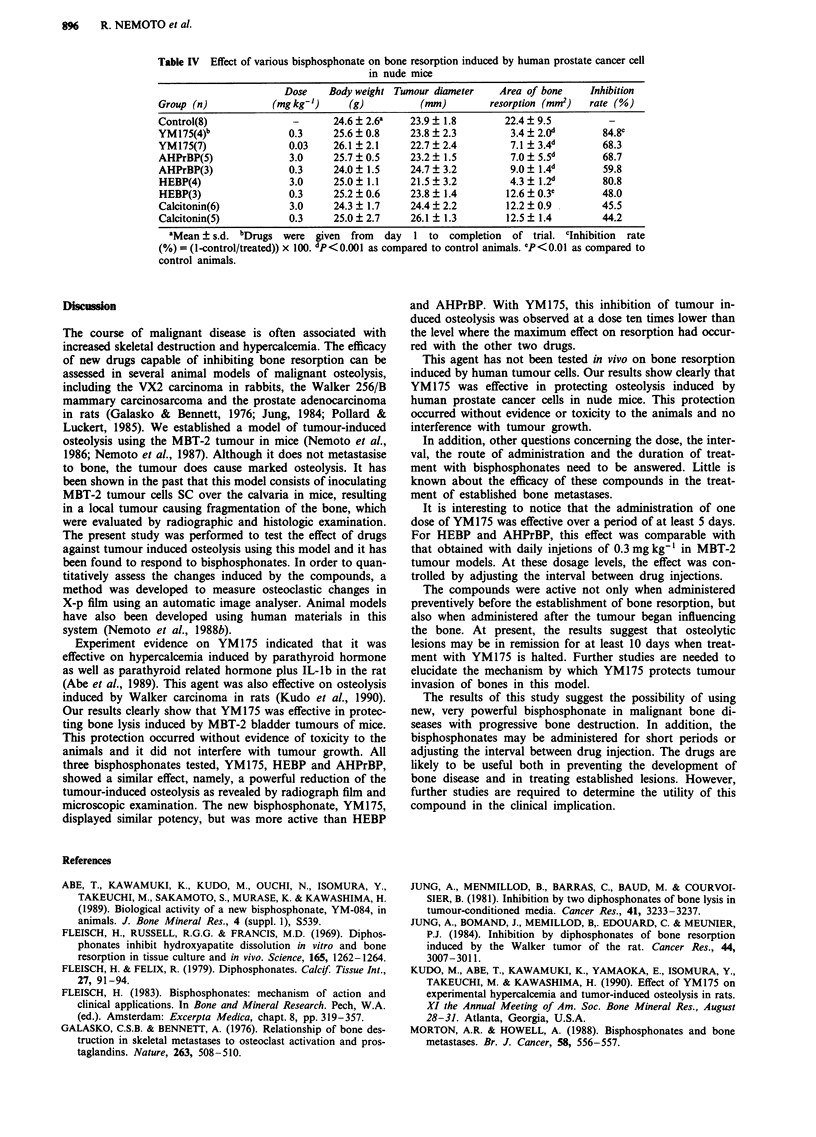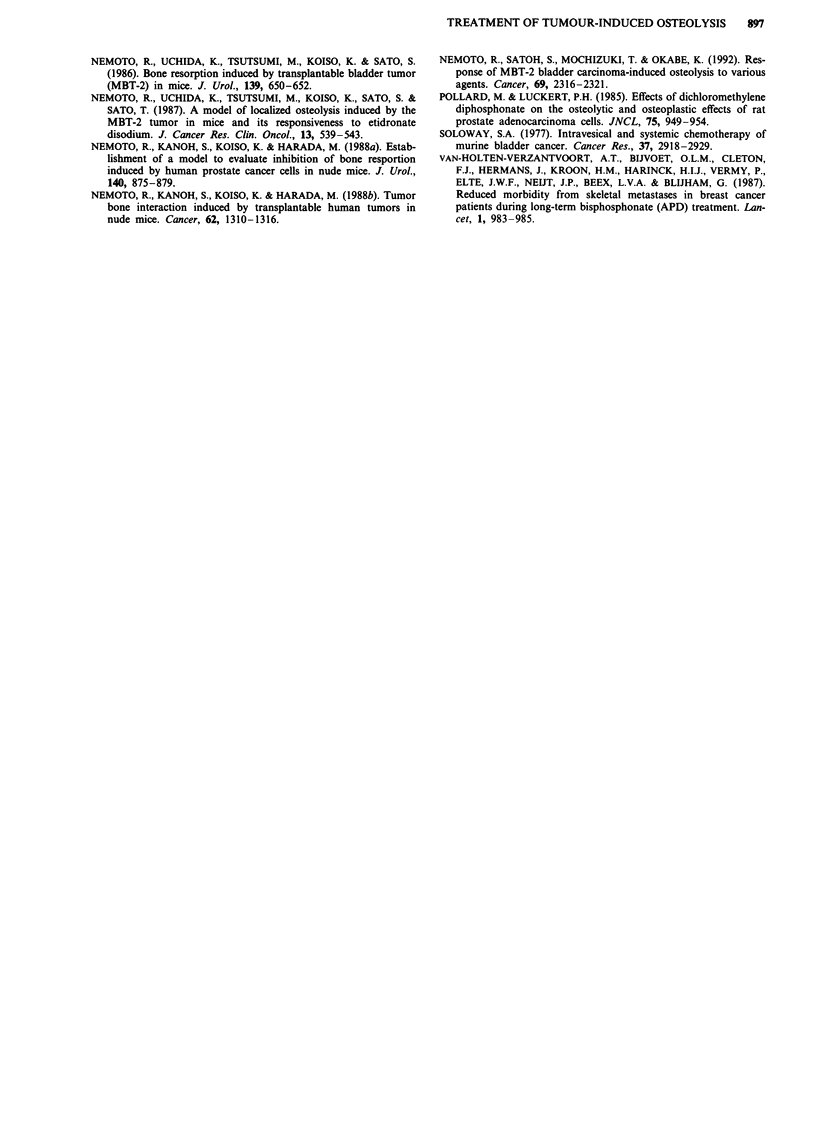# Inhibition by a new bisphosphonate (YM175) of bone resorption induced by the MBT-2 tumour of mice.

**DOI:** 10.1038/bjc.1993.167

**Published:** 1993-05

**Authors:** R. Nemoto, Y. Nishijima, K. Uchida, K. Koiso

**Affiliations:** Department of Urology, Tottori University School of Medicine, Yonago, Japan.

## Abstract

**Images:**


Br. J. Cancer (1993), 67, 893–897 © Macmillan Press Ltd., 1993

### Inhibition by a new bisphosphonate (YM175) of bone resorption induced by the MBT-2 tumour of mice

R. Nemoto[1], Y. Nishijima[2], K. Uchida[2] & K. Koiso[2]

[1]*The Department of Urology, Tottori University School of Medicine, Yonago 683, and* [2]*The Institute of Clinical Medicine, University of Tsukuba, Ibaraki 305, Japan.*

**Summary** A new bisphosphonate, disodium dihydrogen (cycloheptylamino) methylene bisphosphonate monohydrate (YM175), was compared with 3-amino-1-hydroxypropylidene-1, 1-bisphosphonate (AHPrBP) and 1-hydroxyethylidene-1,1-bisphosphonate (HEBP) in terms of its effect on tumour induced osteolysis using a bladder tumour in mice (MBT-2). The method consisted of inoculating tumour cells subcutaneously (SC) over the calvaria in mice, resulting in a local tumour causing fragmentation of the bone. The compounds were active not only when administered preventively before establishment of bone resorption, but also in an inhibitory fashion once the variables were already under the influence of the tumour. This osteolysis was evaluated by measuring the increased area of bone resorption in reduced opacity to radiograph and histology. The results showed the following sequence of potency: YM175 > AHPrBP = HEBP. This inhibition was obtained with no apparent effect on the growth of the MBT-2 tumour. YM175 appears to be an interesting new bisphosphonate with possible clinical application.

There are at least two mechanisms for osteolysis that occurs in both human metastases and experimental skeletal metastasis; osteoclast mediated bone destruction and direct destruction of bone independent of osteoclasts (Galasko & Bennett, 1976). Thus, agents that inhibit bone resorption might be used to decrease the extent of bone destruction by the tumour. Based on their action on calcium phosphate crystal formation and on bone resorption, bisphosphonates represent a new class of drugs with considerable therapeutic potential for metastatic bone disease (Fleisch *et al.*, 1969; Fleisch & Felix, 1979; Fleisch, 1983). Inhibition of tumour induced osteolysis by bisphosphonates has been noted in animal and human studies (Jung *et al.*, 1981; VanHolten-Verzantvoort *et al.*, 1987; Morton & Howell, 1988).

With regard to experimental model systems of neoplasm-associated local osteolysis, tumour-bone interactions using calvaria of the mice were extensively studied by our group (Nemoto *et al.*, 1986; Nemoto *et al.*, 1987; Nemoto *et al.*, 1988*a*; Nemoto *et al.*, 1988*b*). In one of the recent studies, suggestive evidence was obtained that our system might be suitable for studying the biology of local interaction between bone and cancer cells. Furthermore, it is suggested that bone destruction induced by tumour cell invasion involves at least two mechanisms; osteoclast mediated bone destruction, and direct destruction of bone independent of osteoclasts. Bisphosphonate makes bone less susceptible to the action of both osteoclasts and tumour cells (Nemoto *et al.*, 1992).

In this paper, a new more powerful bisphosphonate, YM175 was investigated and compared to both AHPrBP and HEBP using as a model of osteolysis induced by MBT-2 tumour in mice and human prostate cancer cells in nude mice.

### Materials and methods

### *Animals*

Female C3H/He mice, 8 to 12 weeks old, were purchased from Clea Japan, Tokyo. Male BALB/c-nu/nu nude mice, aged 8–12 weeks, were obtained from Clea Japan Inc., Tokyo, and were housed in standard cages fitted with filter tops and handled in laminal flow hoods. They were fed standard irradiated mouse diet and sterile water ad libitum.

Correspondence: R. Nemoto.
Received 30 September 1992; and in revised form 7 December 1992.

### *Tumours*

The tumour utilised in this experiment originated as an invasive transitional cell carcinoma in an inbred C3H/He female mouse having ingested the bladder carcinogen FANFT for 11 months. This tumour, designated MBT-2, was originally provided by Dr M.S. Soloway and has been serially transplanted in syngenic mice (Soloway, 1977). The tumour has retained the histologic appearance of a poorly differentiated transitional cell carcinoma. TSU-PR1 is a cell line derived from a primary adenocarcinoma of the prostate in a 73-year-old man with multiple osteoblastic bone metastases (Nemoto *et al.*, 1988*b*).

### *Induction of osteolysis*

MBT-2 cells were transplanted routinely by subcutaneously (SC) inoculation into female C3H/He mice. When the resulting tumour was visible, it was excised aseptically and minced in medium 199. The tumour mince was further disrupted by repeated syringing. A cell suspension containing $10^6$ tumour cells in 0.2 ml medium was inoculated SC over the calvaria of C3H/He mice under ether anaesthesia. The viability of tumour cells were assessed by using trypan blue dye exclusion test. As the tumour cells were inoculated, the needle was used to scratch the bone, which disrupted the periosteum. The same method was used for the induction of osteolysis in the nude mice using TSU-PR1 human prostate carcinoma.

### *Radiographic and microscopic examination*

Tumour-bearing animals were killed under anaesthesia when the tumour grew to cover the calvaria. Tumours were measured with calipers, and the mean tumour diameter was calculated from the equation $(L + W) \times 0.5$, where L is the major and W the minor diameter. The two diameters were measured at right angles to each other. Mice with tumours less than 10 mm in diameter were excluded from analysis. The parietal bones with attached tumour transplants were fixed in buffered formaline and transferred to ethanol. Radiographs were examined in blind conditions. The area of bone resorption from radiograph detectable lesions was measured by computerised analysis using graphtec software for plotters. After decalcification by EDTA, the same samples were processed for histologic examination.

### *Treatment*

The following bisphosphonates were used: YM175: disodium dihydrogen (cycloheptylamino) methylene bisphosphonate monohydrate, AHPrBP: 3-amino-1-hydroxypropylidene-1,1-bisphosphonate, and HEBP: 1-hydroxyethylidene-1, 1-bisphosphonate. YM175, HEBP and AHPrBP were synthesised by the Department of Pharmacology, Medical Research Laboratories, Central Research Laboratories, Yamanouchi Pharmaceutical Co., Ltd. in Japan. Eel calcitonin (Sigma) was dissolved in 0.05% NaCl and 0.2% sodium acetate in distilled water containing 1 mg $ml^{-1}$ of bovine albumin (Sigma). The bisphosphonates were dissolved in 0.9% NaCl solution and injected SC into the flanks. The doses and schedule are specified in the tables and figures. Control animals were given the solvent.

*Statistical analysis*

The data were analysed by one-way analysis of variance to detect differences between groups. When difference were detected ($P<0.05$), Fisher's multiple range test was used to determine the significance. All values are presented as mean ± s.d.

### Results

If the tumour was not grown or not larger than 10 mm, the mice were excluded from additional evaluation as judged by radiography and histological examination.

The effects of various types of bisphosphonates and calcitonin on bone resorption induced by MBT-2 tumours in mice assessed by graphtec software for plotters in radiographs are presented in Table I. The inhibition rate (%) was calculated from the equation (1-control/treated) × 100. Animals treated with the lower doses of YM175 (0.03 mg $kg^{-1}$) showed weakened adhesion of tumours to skull and bone perforation was infrequently observed. The radiograph detectable bone lesions showed significant reduction of osteolysis ($P<0.001$). Furthermore, YM175 seemed to be remarkably active when used in higher concentrations of 0.3 mg $kg^{-1}$. All bisphosphonates showed a clear dose-related effect. The least active bisphosphonates were AHPrBP and HEBP. The most active compound, YM175, was about more than ten times more active than AHPrBP and HEBP. This inhibitory effect on bone invasion by MBT-2 tumours was not accompanied by a reduction of tumour growth. The mice tolerated the injections well and there was no evidence of toxicity on the basis of body weight, physical appearance or examination of visceral organs. No effect on tumour induced osteolysis was obtained with calcitonin.

The effect of YM175 on bone resorption induced by MBT-2 tumours was influenced by the interval between drug injections (Table II). The administration of 0.3 mg $kg^{-1}$ of YM 175 every 3 days significantly reduced bone resorption ($P<0.01$). Total dose of 6.0 mg $kg^{-1}$ YM-175 was administered by the interval of every 5 days (1.5 mg $kg^{-1}$ $day^{-1}$) and 10 days (3.0 mg $kg^{-1}$ $day^{-1}$). The inhibitory effect was significant.

The compounds were active not only when administered preventively before the establishment of bone resorption, but also when administered after the tumour began influencing the bone (Figure 1a,b,c,d). Table III shows the effects of YM175 injected 14 days after the induction of bone resorption using MBT-2 tumours. The lower concentration of 0.3 mg $kg^{-1}$ of YM175 significantly ($P<0.05$) suppressed osteolysis when compared with the control mice sacrificed on day 23. No effects on tumour induced osteolysis was obtained with other drugs.

The radiographs show slight disconnection of the sagittal suture of calvaria in the treated group in which osteolytic lesions were scarce. Microscopic examinations revealed that the sagittal sutures were divided by tumour invasion through the connective tissue, not by the osteolytic activity.

The effects of various types of bisphosphonates and calcitonin on bone resorption induced by TSU-PR1 tumours in nude mice assessed by graphtec software for plotters in radiographs are presented in Table IV. The radiograph detectable bone lesions showed significant reduction of osteolysis ($P<0.001$). All bisphosphonates showed a clear dose-related effect as same as MBT-2 tumour induced osteolysis. No effect on tumour induced osteolysis was obtained with calcitonin.

**Table I** Effect of various bisphosphonate on bone resorption induced by MBT-2 tumour in mice

| *Group (n)* | *Dose (mg $kg^{-1}$)* | *Body weight (g)* | *Tumour diameter (mm)* | *Area of bone resorption ($mm^2$)* | *Inhibition rate (%)* |
|---|---|---|---|---|---|
| Control(10) | – | 27.6 ± 1.5[a] | 22.2 ± 2.8 | 36.0 ± 14.1 | – |
| YM175(4)[b] | 3.0 | 24.6 ± 0.9 | 22.6 ± 2.1 | 2.0 ± 1.0[d] | 94.5[c] |
| YM175(7) | 0.3 | 26.0 ± 0.7 | 22.6 ± 2.1 | 11.3 ± 3.7[d] | 68.7[c] |
| YM175(6) | 0.03 | 28.8 ± 1.9 | 18.3 ± 1.6 | 16.2 ± 8.0[d] | 55.0 |
| AHPrBP(7) | 3.0 | 24.6 ± 0.7 | 23.4 ± 1.9 | 16.0 ± 3.1[d] | 55.6 |
| AHPrBP(7) | 0.3 | 25.2 ± 1.4 | 23.3 ± 1.9 | 28.8 ± 10.2 | 20.0 |
| HEBP(5) | 3.0 | 26.7 ± 2.6 | 22.6 ± 3.9 | 7.8 ± 6.1[d] | 78.4 |
| HEBP(6) | 0.3 | 27.0 ± 1.0 | 23.4 ± 2.3 | 28.0 ± 6.5 | 22.3 |
| Calcitonin(7) | 3.0 | 25.6 ± 1.0 | 24.0 ± 1.0 | 31.7 ± 8.6 | 12.0 |
| Calcitonin(6) | 0.3 | 27.3 ± 2.8 | 22.7 ± 3.9 | 27.2 ± 13.5 | 24.5 |

[a]Mean ± s.d. [b]Drugs were given from day 1 to completion of trial. [c]Inhibition rate (%) = (1-control/treated) × 100. [d]$P<0.001$ as compared to control animals.

**Table II** Effect of various dose intervals of YM175 on bone resorption induced by MBT-2 tumour in mice

| *Treatment(dose,n)* | *Body weight (g)* | *Tumour diameter (mm)* | *Area of bone resorption ($mm^2$)* |
|---|---|---|---|
| Control(6) | 20.6 ± 1.0[a] | 20.4 ± 4.9 | 34.7 ± 13.6 |
| Interval between drug injections (day)[b] | | | |
| 1 (0.3 mg kg day,8) | 20.4 ± 0.9 | 21.4 ± 3.0 | 9.0 ± 4.6[c] |
| 3 (0.3 mg kg day,7) | 21.4 ± 1.1 | 20.9 ± 3.2 | 18.7 ± 9.9[d] |
| 5 (0.3 mg kg day,6) | 19.6 ± 1.1 | 21.4 ± 2.5 | 26.3 ± 13.9 |
| 10 (0.3 mg kg day,8) | 20.1 ± 0.7 | 20.6 ± 1.7 | 36.8 ± 16.0 |
| 5 (1.5 mg kg day,7) | 20.0 ± 1.3 | 19.7 ± 2.3 | 9.7 ± 2.4[c] |
| 10 (3.0 mg kg day,4) | 20.0 ± 1.8 | 17.5 ± 4.7 | 15.3 ± 6.9[d] |

[a]Mean ± s.d. [b]YM175 was given from day 1 to day 20. Mice were sacrificed day 22. [c]$P<0.001$ as compared to control animals. [d]$P<0.01$ as compared to control animals.

**Figure 1** Radiographs of mouse calvaria. **a**, Control without tumour growth, inoculated subcutaneously over the calvaria with MBT-2 tumour cells after scratching the surface of the bone and sacrificed day 22. **b**, Non-treated control, inoculated subcutaneously over the calvaria with MBT-2 tumour cells after scratching the surface of the bone and sacrificed day 14. Several punched out lesions of the skull are shown. **c**, Nontreated control, inoculated subcutaneously over the calvaria with MBT-2 tumour cells after scratching the surface of the bone and sacrificed day 22. Marked bone destruction is evident. **d**, Calvaria from mouse treated with 3.0 mg $kg^{-1}$ of YM175 from day 14 to day 21. Mice were sacrificed day 22. Osteolysis and punched out lesions are suppressed as mice sacrificed day 14.

**Table III** Effect of YM175 on prior establishment of bone resorption induced MBT-2 tumour in mice

| Group | *Dose(n) (mg kg⁻¹)* | *Body weight (g)* | *Tumour diameter (mm)* | *Area of bone resorption (mm²)* |
|---|---|---|---|---|
| Control | | | | |
| Sacrifice at day | 14(11) | 21.5 ± 0.9[a] | 9.3 ± 3.9 | 23.4 ± 11.9 |
| Sacrifice at day | 22(10) | 21.8 ± 1.1 | 20.8 ± 5.0 | 53.6 ± 14.1 |
| YM175[b] | | | | |
| | 3.0 mg $kg^{-1}$(7) | 21.8 ± 1.1 | 22.1 ± 4.4 | 27.3 ± 10.8[c] |
| | 0.3 mg $kg^{-1}$(6) | 22.3 ± 0.8 | 21.8 ± 4.7 | 36.2 ± 9.2[d] |
| AHPrBP[b] | | | | |
| | 3.0 mg $kg^{-1}$(6) | 21.9 ± 1.5 | 21.1 ± 4.1 | 44.2 ± 15.8 |
| | 0.3 mg $kg^{-1}$(4) | 21.9 ± 1.3 | 21.9 ± 5.2 | 57.8 ± 8.4 |
| HEBP[b] | | | | |
| | 3.0 mg $kg^{-1}$(8) | 21.6 ± 1.4 | 22.0 ± 3.8 | 44.3 ± 17.0 |
| | 0.3 mg $kg^{-1}$(7) | 22.4 ± 1.1 | 20.9 ± 4.3 | 60.1 ± 5.6 |
| Calcitonin[b] | | | | |
| | 3.0 mg $kg^{-1}$(5) | 21.8 ± 0.8 | 19.3 ± 3.0 | 40.3 ± 24.6 |
| | 0.3 mg $kg^{-1}$(6) | 21.1 ± 1.7 | 21.2 ± 4.1 | 53.2 ± 10.6 |

[a]Mean ± standard deviation. [b]Drugs were given from day 14 to completion of trial and mice were sacrificed at day 22. [c]$P<0.01$ as compared with control animals. [d]$P<0.05$ as compared with control animals.

**Table IV** Effect of various bisphosphonate on bone resorption induced by human prostate cancer cell in nude mice

| *Group (n)* | *Dose ($mg\,kg^{-1}$)* | *Body weight (g)* | *Tumour diameter (mm)* | *Area of bone resorption ($mm^2$)* | *Inhibition rate (%)* |
|---|---|---|---|---|---|
| Control(8) | – | 24.6 ± 2.6[a] | 23.9 ± 1.8 | 22.4 ± 9.5 | – |
| YM175(4)[b] | 0.3 | 25.6 ± 0.8 | 23.8 ± 2.3 | 3.4 ± 2.0[d] | 84.8[c] |
| YM175(7) | 0.03 | 26.1 ± 2.1 | 22.7 ± 2.4 | 7.1 ± 3.4[d] | 68.3 |
| AHPrBP(5) | 3.0 | 25.7 ± 0.5 | 23.2 ± 1.5 | 7.0 ± 5.5[d] | 68.7 |
| AHPrBP(3) | 0.3 | 24.0 ± 1.5 | 24.7 ± 3.2 | 9.0 ± 1.4[d] | 59.8 |
| HEBP(4) | 3.0 | 25.0 ± 1.1 | 21.5 ± 3.2 | 4.3 ± 1.2[d] | 80.8 |
| HEBP(3) | 0.3 | 25.2 ± 0.6 | 23.8 ± 1.4 | 12.6 ± 0.3[e] | 48.0 |
| Calcitonin(6) | 3.0 | 24.3 ± 1.7 | 24.4 ± 2.2 | 12.2 ± 0.9 | 45.5 |
| Calcitonin(5) | 0.3 | 25.0 ± 2.7 | 26.1 ± 1.3 | 12.5 ± 1.4 | 44.2 |

[a]Mean ± s.d. [b]Drugs were given from day 1 to completion of trial. [c]Inhibition rate (%) = (1-control/treated)) × 100. [d]$P<0.001$ as compared to control animals. [e]$P<0.01$ as compared to control animals.

### Discussion

The course of malignant disease is often associated with increased skeletal destruction and hypercalcemia. The efficacy of new drugs capable of inhibiting bone resorption can be assessed in several animal models of malignant osteolysis, including the VX2 carcinoma in rabbits, the Walker 256/B mammary carcinosarcoma and the prostate adenocarcinoma in rats (Galasko & Bennett, 1976; Jung, 1984; Pollard & Luckert, 1985). We established a model of tumour-induced osteolysis using the MBT-2 tumour in mice (Nemoto *et al.*, 1986; Nemoto *et al.*, 1987). Although it does not metastasise to bone, the tumour does cause marked osteolysis. It has been shown in the past that this model consists of inoculating MBT-2 tumour cells SC over the calvaria in mice, resulting in a local tumour causing fragmentation of the bone, which were evaluated by radiographic and histologic examination. The present study was performed to test the effect of drugs against tumour induced osteolysis using this model and it has been found to respond to bisphosphonates. In order to quantitatively assess the changes induced by the compounds, a method was developed to measure osteoclastic changes in X-p film using an automatic image analyser. Animal models have also been developed using human materials in this system (Nemoto *et al.*, 1988*b*).

Experiment evidence on YM175 indicated that it was effective on hypercalcemia induced by parathyroid hormone as well as parathyroid related hormone plus IL-1b in the rat (Abe *et al.*, 1989). This agent was also effective on osteolysis induced by Walker carcinoma in rats (Kudo *et al.*, 1990). Our results clearly show that YM175 was effective in protecting bone lysis induced by MBT-2 bladder tumours of mice. This protection occurred without evidence of toxicity to the animals and it did not interfere with tumour growth. All three bisphosphonates tested, YM175, HEBP and AHPrBP, showed a similar effect, namely, a powerful reduction of the tumour-induced osteolysis as revealed by radiograph film and microscopic examination. The new bisphosphonate, YM175, displayed similar potency, but was more active than HEBP and AHPrBP. With YM175, this inhibition of tumour induced osteolysis was observed at a dose ten times lower than the level where the maximum effect on resorption had occurred with the other two drugs.

This agent has not been tested *in vivo* on bone resorption induced by human tumour cells. Our results show clearly that YM175 was effective in protecting osteolysis induced by human prostate cancer cells in nude mice. This protection occurred without evidence or toxicity to the animals and no interference with tumour growth.

In addition, other questions concerning the dose, the interval, the route of administration and the duration of treatment with bisphosphonates need to be answered. Little is known about the efficacy of these compounds in the treatment of established bone metastases.

It is interesting to notice that the administration of one dose of YM175 was effective over a period of at least 5 days. For HEBP and AHPrBP, this effect was comparable with that obtained with daily injetions of $0.3\,mg\,kg^{-1}$ in MBT-2 tumour models. At these dosage levels, the effect was controlled by adjusting the interval between drug injections.

The compounds were active not only when administered preventively before the establishment of bone resorption, but also when administered after the tumour began influencing the bone. At present, the results suggest that osteolytic lesions may be in remission for at least 10 days when treatment with YM175 is halted. Further studies are needed to elucidate the mechanism by which YM175 protects tumour invasion of bones in this model.

The results of this study suggest the possibility of using new, very powerful bisphosphonate in malignant bone diseases with progressive bone destruction. In addition, the bisphosphonates may be administered for short periods or adjusting the interval between drug injection. The drugs are likely to be useful both in preventing the development of bone disease and in treating established lesions. However, further studies are required to determine the utility of this compound in the clinical implication.

### References

ABE, T., KAWAMUKI, K., KUDO, M., OUCHI, N., ISOMURA, Y., TAKEUCHI, M., SAKAMOTO, S., MURASE, K. & KAWASHIMA, H. (1989). Biological activity of a new bisphosphonate, YM-084, in animals. *J. Bone Mineral Res.*, **4** (suppl. 1), S539.

FLEISCH, H., RUSSELL, R.G.G. & FRANCIS, M.D. (1969). Diphosphonates inhibit hydroxyapatite dissolution *in vitro* and bone resorption in tissue culture and *in vivo*. *Science*, **165**, 1262–1264.

FLEISCH, H. & FELIX, R. (1979). Diphosphonates. *Calcif. Tissue Int.*, **27**, 91–94.

FLEISCH, H. (1983). Bisphosphonates: mechanism of action and clinical applications. In *Bone and Mineral Research*. Pech, W.A. (ed.). Amsterdam: *Excerpta Medica*, chapt. 8, pp. 319–357.

GALASKO, C.S.B. & BENNETT, A. (1976). Relationship of bone destruction in skeletal metastases to osteoclast activation and prostaglandins. *Nature*, **263**, 508–510.

JUNG, A., MENMILLOD, B., BARRAS, C., BAUD, M. & COURVOISIER, B. (1981). Inhibition by two diphosphonates of bone lysis in tumour-conditioned media. *Cancer Res.*, **41**, 3233–3237.

JUNG, A., BOMAND, J., MEMILLOD, B,. EDOUARD, C. & MEUNIER, P.J. (1984). Inhibition by diphosphonates of bone resorption induced by the Walker tumor of the rat. *Cancer Res.*, **44**, 3007–3011.

KUDO, M., ABE, T., KAWAMUKI, K., YAMAOKA, E., ISOMURA, Y., TAKEUCHI, M. & KAWASHIMA, H. (1990). Effect of YM175 on experimental hypercalcemia and tumor-induced osteolysis in rats. *XI the Annual Meeting of Am. Soc. Bone Mineral Res., August 28–31*. Atlanta, Georgia, U.S.A.

MORTON, A.R. & HOWELL, A. (1988). Bisphosphonates and bone metastases. *Br. J. Cancer*, **58**, 556–557.

NEMOTO, R., UCHIDA, K., TSUTSUMI, M., KOISO, K. & SATO, S. (1986). Bone resorption induced by transplantable bladder tumor (MBT-2) in mice. *J. Urol.*, **139**, 650–652.

NEMOTO, R., UCHIDA, K., TSUTSUMI, M., KOISO, K., SATO, S. & SATO, T. (1987). A model of localized osteolysis induced by the MBT-2 tumor in mice and its responsiveness to etidronate disodium. *J. Cancer Res. Clin. Oncol.*, **13**, 539–543.

NEMOTO, R., KANOH, S., KOISO, K. & HARADA, M. (1988*a*). Establishment of a model to evaluate inhibition of bone resportion induced by human prostate cancer cells in nude mice. *J. Urol.*, **140**, 875–879.

NEMOTO, R., KANOH, S., KOISO, K. & HARADA, M. (1988*b*). Tumor bone interaction induced by transplantable human tumors in nude mice. *Cancer*, **62**, 1310–1316.

NEMOTO, R., SATOH, S., MOCHIZUKI, T. & OKABE, K. (1992). Response of MBT-2 bladder carcinoma-induced osteolysis to various agents. *Cancer*, **69**, 2316–2321.

POLLARD, M. & LUCKERT, P.H. (1985). Effects of dichloromethylene diphosphonate on the osteolytic and osteoplastic effects of rat prostate adenocarcinoma cells. *JNCL*, **75**, 949–954.

SOLOWAY, S.A. (1977). Intravesical and systemic chemotherapy of murine bladder cancer. *Cancer Res.*, **37**, 2918–2929.

VAN-HOLTEN-VERZANTVOORT, A.T., BIJVOET, O.L.M., CLETON, F.J., HERMANS, J., KROON, H.M., HARINCK, H.I.J., VERMY, P., ELTE, J.W.F., NEIJT, J.P., BEEX, L.V.A. & BLIJHAM, G. (1987). Reduced morbidity from skeletal metastases in breast cancer patients during long-term bisphosphonate (APD) treatment. *Lancet*, **1**, 983–985.